# Supplementary material for: Hot spot prediction in protein-protein interactions by an ensemble system
Source: BMC Syst Biol. 2018 Dec 31;12(Suppl 9):132. doi: 10.1186/s12918-018-0665-8 (PMC6311905; doi:10.1186/s12918-018-0665-8)
Supplement: Supplementary file 2 — All relevant MATLAB code. A simple Matlab implement of our predictor. (ZIP 19,742 kb) [file 12918_2018_665_MOESM2_ESM.zip › Additional file 2/Read ASA/ASA website/dbMPIKT/4YEBB.html]

 NetSurfP - Protein Surface Accessibility and Secondary Structure Predictions

  

|  |  |  |
| --- | --- | --- |
|  |  | NetSurfP - Protein Surface Accessibility and Secondary Structure Predictions  Technical University of Denmark |

  


---

```
# For publication of results, please cite:
# A generic method for assignment of reliability scores applied to solvent accessibility predictions.
# Bent Petersen, Thomas Nordahl Petersen, Pernille Andersen, Morten Nielsen and Claus Lundegaard
# BMC Structural Biology 2009, 9:51 doi:10.1186/1472-6807-9-51
#
# Column 1: Class assignment - B for buried or E for Exposed - Threshold: 25% exposure, but not based on RSA
# Column 2: Amino acid
# Column 3: Sequence name
# Column 4: Amino acid number
# Column 5: Relative Surface Accessibility - RSA
# Column 6: Absolute Surface Accessibility
# Column 7: Z-fit score for RSA prediction
# Column 8: Probability for Alpha-Helix
# Column 9: Probability for Beta-strand
# Column 10: Probability for Coil
E A  4YEBB                  1    0.837  92.226  -2.120   0.003   0.003   0.994
E P  4YEBB                  2    0.469  66.622  -2.138   0.005   0.015   0.979
E S  4YEBB                  3    0.411  48.111  -2.049   0.005   0.015   0.979
E T  4YEBB                  4    0.567  78.712  -2.551   0.018   0.019   0.964
E D  4YEBB                  5    0.643  92.599  -0.933   0.016   0.005   0.979
E P  4YEBB                  6    0.469  66.509  -0.901   0.018   0.019   0.964
E K  4YEBB                  7    0.605 124.407  -0.106   0.018   0.019   0.964
E S  4YEBB                  8    0.517  60.604   0.418   0.018   0.019   0.964
B C  4YEBB                  9    0.048   6.739  -0.002   0.018   0.019   0.964
B P  4YEBB                 10    0.171  24.222  -0.068   0.018   0.019   0.964
E S  4YEBB                 11    0.659  77.258  -0.310   0.018   0.019   0.964
E V  4YEBB                 12    0.510  78.448   0.323   0.018   0.019   0.964
B C  4YEBB                 13    0.042   5.869  -1.472   0.018   0.088   0.893
E R  4YEBB                 14    0.449 102.867  -0.036   0.005   0.262   0.733
B C  4YEBB                 15    0.115  16.118  -0.880   0.004   0.197   0.799
E D  4YEBB                 16    0.471  67.842   0.469   0.004   0.197   0.799
B A  4YEBB                 17    0.246  27.164  -0.785   0.005   0.336   0.660
E G  4YEBB                 18    0.434  34.164   0.668   0.003   0.718   0.279
E F  4YEBB                 19    0.356  71.550   1.223   0.001   0.959   0.040
B I  4YEBB                 20    0.040   7.363   0.472   0.001   0.959   0.040
B Y  4YEBB                 21    0.259  55.263   0.511   0.001   0.900   0.099
B C  4YEBB                 22    0.038   5.279   0.053   0.004   0.616   0.381
E N  4YEBB                 23    0.368  53.817  -0.200   0.005   0.262   0.733
E D  4YEBB                 24    0.631  90.898  -0.792   0.005   0.045   0.951
B R  4YEBB                 25    0.283  64.830   0.473   0.018   0.047   0.935
E S  4YEBB                 26    0.481  56.385   0.773   0.018   0.088   0.893
B L  4YEBB                 27    0.050   9.210  -0.568   0.019   0.141   0.840
E T  4YEBB                 28    0.489  67.880   1.096   0.021   0.279   0.699
E S  4YEBB                 29    0.507  59.432   1.483   0.020   0.205   0.775
B I  4YEBB                 30    0.063  11.674   1.176   0.018   0.088   0.893
E P  4YEBB                 31    0.241  34.212   0.930   0.018   0.019   0.964
E V  4YEBB                 32    0.675 103.686   0.435   0.176   0.004   0.820
E G  4YEBB                 33    0.645  50.793  -0.356   0.246   0.004   0.750
B I  4YEBB                 34    0.121  22.348   0.486   0.321   0.003   0.675
B P  4YEBB                 35    0.089  12.587  -0.751   0.321   0.003   0.675
E E  4YEBB                 36    0.619 108.139   1.142   0.430   0.016   0.555
E D  4YEBB                 37    0.418  60.219   1.163   0.430   0.016   0.555
B A  4YEBB                 38    0.044   4.893   0.559   0.386   0.097   0.517
E T  4YEBB                 39    0.329  45.660   1.298   0.231   0.330   0.439
E T  4YEBB                 40    0.389  53.968   1.812   0.085   0.775   0.140
B L  4YEBB                 41    0.034   6.299   0.692   0.072   0.855   0.073
B Y  4YEBB                 42    0.256  54.750   0.764   0.085   0.775   0.140
B L  4YEBB                 43    0.036   6.647  -0.653   0.021   0.279   0.699
E Q  4YEBB                 44    0.399  71.172   0.226   0.019   0.141   0.840
E N  4YEBB                 45    0.512  75.030  -0.153   0.018   0.047   0.935
B N  4YEBB                 46    0.082  11.946  -0.868   0.018   0.088   0.893
E Q  4YEBB                 47    0.428  76.387   0.754   0.020   0.205   0.775
B I  4YEBB                 48    0.045   8.399  -1.700   0.020   0.205   0.775
E N  4YEBB                 49    0.475  69.511   0.201   0.020   0.205   0.775
E N  4YEBB                 50    0.595  87.137  -0.666   0.004   0.197   0.799
B V  4YEBB                 51    0.159  24.454   0.190   0.004   0.138   0.858
E G  4YEBB                 52    0.654  51.493  -1.334   0.004   0.085   0.910
E I  4YEBB                 53    0.327  60.421  -0.083   0.058   0.017   0.925
E P  4YEBB                 54    0.360  51.098  -0.707   0.246   0.004   0.750
E S  4YEBB                 55    0.682  79.954  -0.597   0.321   0.003   0.675
E D  4YEBB                 56    0.277  39.901  -0.328   0.321   0.003   0.675
B L  4YEBB                 57    0.075  13.751  -1.747   0.339   0.016   0.645
E K  4YEBB                 58    0.460  94.643   0.151   0.257   0.016   0.727
E N  4YEBB                 59    0.605  88.631   0.529   0.109   0.005   0.886
B L  4YEBB                 60    0.072  13.147   0.463   0.018   0.019   0.964
E L  4YEBB                 61    0.517  94.718   0.749   0.018   0.019   0.964
E K  4YEBB                 62    0.464  95.548   1.533   0.018   0.047   0.935
B V  4YEBB                 63    0.059   9.053   0.753   0.018   0.088   0.893
E Q  4YEBB                 64    0.351  62.617   1.337   0.021   0.279   0.699
E R  4YEBB                 65    0.391  89.447   1.688   0.011   0.918   0.071
B I  4YEBB                 66    0.038   7.104   0.584   0.011   0.918   0.071
E Y  4YEBB                 67    0.295  63.106   0.731   0.018   0.846   0.136
B L  4YEBB                 68    0.041   7.507  -0.377   0.021   0.279   0.699
E Y  4YEBB                 69    0.390  83.300  -0.241   0.018   0.088   0.893
E H  4YEBB                 70    0.578 105.156  -0.189   0.018   0.047   0.935
B N  4YEBB                 71    0.110  16.163  -0.840   0.018   0.047   0.935
E S  4YEBB                 72    0.428  50.162   1.023   0.021   0.279   0.699
B L  4YEBB                 73    0.039   7.232  -1.067   0.022   0.359   0.619
E D  4YEBB                 74    0.470  67.741   0.592   0.022   0.359   0.619
E E  4YEBB                 75    0.473  82.616   0.216   0.020   0.205   0.775
B F  4YEBB                 76    0.095  19.147   1.178   0.018   0.047   0.935
E P  4YEBB                 77    0.286  40.569   0.678   0.115   0.016   0.868
E T  4YEBB                 78    0.664  92.041  -0.053   0.502   0.002   0.495
E N  4YEBB                 79    0.601  87.986  -0.904   0.406   0.004   0.590
B L  4YEBB                 80    0.106  19.317  -0.487   0.502   0.002   0.495
B P  4YEBB                 81    0.077  10.969  -1.416   0.430   0.016   0.555
E K  4YEBB                 82    0.602 123.831   1.266   0.430   0.016   0.555
E Y  4YEBB                 83    0.350  74.816   0.852   0.430   0.016   0.555
B V  4YEBB                 84    0.049   7.485   0.616   0.354   0.048   0.598
E K  4YEBB                 85    0.336  69.156   1.263   0.216   0.235   0.548
E E  4YEBB                 86    0.400  69.880   1.800   0.172   0.690   0.138
B L  4YEBB                 87    0.037   6.848   0.491   0.085   0.775   0.140
E H  4YEBB                 88    0.284  51.660   1.226   0.085   0.775   0.140
B L  4YEBB                 89    0.039   7.068  -0.408   0.066   0.296   0.638
E Q  4YEBB                 90    0.385  68.672  -0.079   0.019   0.141   0.840
E E  4YEBB                 91    0.565  98.740  -0.240   0.018   0.047   0.935
B N  4YEBB                 92    0.091  13.308  -0.776   0.018   0.088   0.893
E N  4YEBB                 93    0.447  65.485   0.858   0.020   0.205   0.775
B I  4YEBB                 94    0.052   9.712  -0.157   0.021   0.279   0.699
E R  4YEBB                 95    0.455 104.081   0.668   0.005   0.336   0.660
E T  4YEBB                 96    0.507  70.363  -0.151   0.004   0.197   0.799
B I  4YEBB                 97    0.142  26.214   0.949   0.018   0.047   0.935
E T  4YEBB                 98    0.338  46.839   0.641   0.018   0.019   0.964
E Y  4YEBB                 99    0.497 106.230   0.292   0.694   0.003   0.303
E D  4YEBB                100    0.632  91.114   0.007   0.694   0.003   0.303
B S  4YEBB                101    0.134  15.728  -0.166   0.694   0.003   0.303
B L  4YEBB                102    0.068  12.524  -1.781   0.694   0.003   0.303
E S  4YEBB                103    0.510  59.760   0.648   0.502   0.002   0.495
E K  4YEBB                104    0.564 116.015   0.912   0.246   0.004   0.750
B I  4YEBB                105    0.044   8.122   0.183   0.109   0.005   0.886
E P  4YEBB                106    0.554  78.627   1.184   0.115   0.016   0.868
E Y  4YEBB                107    0.422  90.160   1.701   0.113   0.043   0.844
B L  4YEBB                108    0.050   9.118   0.619   0.052   0.084   0.864
E E  4YEBB                109    0.265  46.365   1.048   0.020   0.205   0.775
E E  4YEBB                110    0.380  66.438   1.797   0.018   0.846   0.136
B L  4YEBB                111    0.028   5.182   0.939   0.011   0.918   0.071
B H  4YEBB                112    0.239  43.383   0.709   0.018   0.846   0.136
B L  4YEBB                113    0.037   6.683  -0.722   0.021   0.451   0.528
B D  4YEBB                114    0.299  43.043  -0.529   0.020   0.205   0.775
E D  4YEBB                115    0.516  74.384  -0.279   0.005   0.045   0.951
B N  4YEBB                116    0.060   8.784  -0.976   0.019   0.141   0.840
E S  4YEBB                117    0.415  48.626   1.020   0.021   0.451   0.528
B V  4YEBB                118    0.039   6.010  -1.379   0.021   0.451   0.528
E S  4YEBB                119    0.403  47.278  -0.077   0.022   0.359   0.619
E A  4YEBB                120    0.557  61.370  -1.546   0.005   0.262   0.733
B V  4YEBB                121    0.217  33.368  -0.107   0.005   0.262   0.733
E S  4YEBB                122    0.416  48.790  -1.405   0.004   0.138   0.858
B I  4YEBB                123    0.231  42.717  -0.701   0.018   0.088   0.893
E E  4YEBB                124    0.437  76.291  -0.586   0.115   0.016   0.868
E E  4YEBB                125    0.495  86.511  -1.700   0.321   0.003   0.675
E G  4YEBB                126    0.477  37.548  -1.874   0.406   0.004   0.590
B A  4YEBB                127    0.150  16.508  -1.246   0.406   0.004   0.590
B F  4YEBB                128    0.115  23.020  -2.597   0.430   0.016   0.555
E R  4YEBB                129    0.519 118.737   0.003   0.246   0.004   0.750
E D  4YEBB                130    0.559  80.552   0.072   0.053   0.005   0.942
B S  4YEBB                131    0.048   5.684  -0.072   0.016   0.005   0.979
E N  4YEBB                132    0.535  78.251   0.794   0.058   0.017   0.925
E Y  4YEBB                133    0.366  78.257   1.468   0.053   0.043   0.903
B L  4YEBB                134    0.043   7.800   0.330   0.053   0.043   0.903
E R  4YEBB                135    0.319  72.982   1.088   0.019   0.141   0.840
E L  4YEBB                136    0.378  69.285   1.681   0.021   0.756   0.223
B L  4YEBB                137    0.035   6.335   0.603   0.018   0.846   0.136
E F  4YEBB                138    0.274  55.032   0.753   0.021   0.756   0.223
B L  4YEBB                139    0.036   6.518  -0.757   0.021   0.279   0.699
E S  4YEBB                140    0.344  40.364  -0.370   0.018   0.088   0.893
E R  4YEBB                141    0.566 129.706  -0.381   0.018   0.019   0.964
B N  4YEBB                142    0.085  12.503  -0.726   0.018   0.047   0.935
E H  4YEBB                143    0.407  73.997   0.832   0.020   0.205   0.775
B L  4YEBB                144    0.041   7.507  -0.698   0.066   0.296   0.638
E S  4YEBB                145    0.404  47.325   0.857   0.021   0.279   0.699
E T  4YEBB                146    0.444  61.541   0.916   0.020   0.205   0.775
B I  4YEBB                147    0.075  13.820   0.918   0.052   0.084   0.864
E P  4YEBB                148    0.241  34.170   0.761   0.115   0.016   0.868
E G  4YEBB                149    0.654  51.486   0.057   0.321   0.003   0.675
E G  4YEBB                150    0.581  45.740  -1.007   0.246   0.004   0.750
B L  4YEBB                151    0.186  34.093  -0.010   0.321   0.003   0.675
B P  4YEBB                152    0.079  11.267  -1.180   0.321   0.003   0.675
E R  4YEBB                153    0.599 137.125   1.126   0.257   0.016   0.727
E T  4YEBB                154    0.352  48.878   0.763   0.257   0.016   0.727
B I  4YEBB                155    0.060  11.156   0.948   0.268   0.043   0.689
E E  4YEBB                156    0.305  53.249   0.920   0.125   0.227   0.648
E E  4YEBB                157    0.403  70.387   1.821   0.085   0.775   0.140
B L  4YEBB                158    0.032   5.804   0.511   0.072   0.855   0.073
E R  4YEBB                159    0.269  61.578   1.113   0.085   0.775   0.140
B L  4YEBB                160    0.035   6.427  -0.914   0.021   0.279   0.699
E D  4YEBB                161    0.426  61.444  -0.027   0.019   0.141   0.840
E D  4YEBB                162    0.555  79.947  -0.175   0.018   0.047   0.935
B N  4YEBB                163    0.077  11.200  -1.352   0.018   0.088   0.893
E R  4YEBB                164    0.400  91.531   0.887   0.021   0.279   0.699
B I  4YEBB                165    0.048   8.880  -1.135   0.005   0.336   0.660
E S  4YEBB                166    0.449  52.646   0.515   0.005   0.336   0.660
E T  4YEBB                167    0.514  71.320  -0.192   0.005   0.262   0.733
B I  4YEBB                168    0.117  21.608   0.903   0.004   0.085   0.910
E S  4YEBB                169    0.343  40.258   0.256   0.018   0.019   0.964
E S  4YEBB                170    0.541  63.429   0.128   0.694   0.003   0.303
E P  4YEBB                171    0.650  92.249  -0.448   0.600   0.003   0.397
B S  4YEBB                172    0.164  19.209  -0.571   0.600   0.003   0.397
B L  4YEBB                173    0.063  11.572  -1.735   0.600   0.003   0.397
E H  4YEBB                174    0.511  92.951   0.374   0.406   0.004   0.590
E G  4YEBB                175    0.601  47.338   0.779   0.176   0.004   0.820
B L  4YEBB                176    0.053   9.704   0.764   0.058   0.017   0.925
E T  4YEBB                177    0.498  69.142   0.729   0.053   0.043   0.903
E S  4YEBB                178    0.401  46.985   1.676   0.018   0.088   0.893
B L  4YEBB                179    0.046   8.404   0.352   0.018   0.088   0.893
E K  4YEBB                180    0.341  70.103   1.113   0.020   0.205   0.775
E R  4YEBB                181    0.371  84.867   1.771   0.011   0.918   0.071
B L  4YEBB                182    0.031   5.603   1.032   0.011   0.918   0.071
B V  4YEBB                183    0.237  36.458   0.825   0.011   0.918   0.071
B L  4YEBB                184    0.031   5.658  -0.220   0.022   0.552   0.426
E D  4YEBB                185    0.316  45.593  -0.407   0.020   0.205   0.775
E G  4YEBB                186    0.495  38.949  -0.487   0.004   0.085   0.910
B N  4YEBB                187    0.061   8.901  -0.694   0.019   0.141   0.840
E L  4YEBB                188    0.401  73.386   0.748   0.022   0.359   0.619
B L  4YEBB                189    0.043   7.837  -1.602   0.022   0.359   0.619
E N  4YEBB                190    0.401  58.721  -0.058   0.022   0.359   0.619
E N  4YEBB                191    0.587  85.908  -1.683   0.005   0.262   0.733
E H  4YEBB                192    0.351  63.938  -0.368   0.004   0.197   0.799
E G  4YEBB                193    0.528  41.554  -1.855   0.004   0.138   0.858
B L  4YEBB                194    0.181  33.178  -0.750   0.018   0.047   0.935
E G  4YEBB                195    0.366  28.788  -0.398   0.058   0.017   0.925
E D  4YEBB                196    0.460  66.286  -0.942   0.406   0.004   0.590
E K  4YEBB                197    0.596 122.535  -0.748   0.502   0.002   0.495
B V  4YEBB                198    0.173  26.636  -0.918   0.502   0.002   0.495
B F  4YEBB                199    0.056  11.319  -1.012   0.522   0.016   0.462
E F  4YEBB                200    0.511 102.538   0.378   0.321   0.003   0.675
E N  4YEBB                201    0.551  80.681   0.655   0.176   0.004   0.820
B L  4YEBB                202    0.047   8.679   0.162   0.053   0.005   0.942
E V  4YEBB                203    0.523  80.416   1.074   0.115   0.016   0.868
E N  4YEBB                204    0.381  55.734   1.715   0.113   0.043   0.844
B L  4YEBB                205    0.040   7.251   0.701   0.053   0.043   0.903
E T  4YEBB                206    0.343  47.616   1.127   0.052   0.084   0.864
E E  4YEBB                207    0.398  69.566   1.654   0.021   0.756   0.223
B L  4YEBB                208    0.034   6.207   0.761   0.018   0.846   0.136
B S  4YEBB                209    0.262  30.695   0.432   0.021   0.756   0.223
B L  4YEBB                210    0.047   8.679  -0.248   0.021   0.279   0.699
E V  4YEBB                211    0.356  54.748  -0.247   0.018   0.088   0.893
E R  4YEBB                212    0.566 129.683  -0.348   0.018   0.047   0.935
B N  4YEBB                213    0.081  11.858  -0.920   0.018   0.088   0.893
E S  4YEBB                214    0.418  48.990   1.146   0.021   0.279   0.699
B L  4YEBB                215    0.042   7.672  -0.856   0.022   0.359   0.619
E T  4YEBB                216    0.385  53.386   0.788   0.022   0.359   0.619
E A  4YEBB                217    0.446  49.138   0.790   0.022   0.359   0.619
B A  4YEBB                218    0.047   5.135   0.923   0.019   0.141   0.840
E P  4YEBB                219    0.238  33.786   0.734   0.053   0.043   0.903
E V  4YEBB                220    0.671 103.133  -0.357   0.430   0.016   0.555
E N  4YEBB                221    0.553  81.032  -1.321   0.321   0.003   0.675
B L  4YEBB                222    0.144  26.293  -0.671   0.246   0.004   0.750
E P  4YEBB                223    0.341  48.402  -1.569   0.176   0.004   0.820
B G  4YEBB                224    0.259  20.360  -0.344   0.176   0.004   0.820
E T  4YEBB                225    0.508  70.501   0.468   0.181   0.016   0.803
E S  4YEBB                226    0.355  41.653   1.111   0.115   0.016   0.868
B L  4YEBB                227    0.042   7.690   0.123   0.113   0.043   0.844
E R  4YEBB                228    0.336  76.990   1.138   0.056   0.142   0.802
E K  4YEBB                229    0.390  80.141   1.842   0.021   0.756   0.223
B L  4YEBB                230    0.032   5.896   0.926   0.011   0.918   0.071
B Y  4YEBB                231    0.251  53.617   0.785   0.018   0.846   0.136
B L  4YEBB                232    0.039   7.214  -0.375   0.022   0.359   0.619
E Q  4YEBB                233    0.369  65.886  -0.405   0.018   0.088   0.893
E D  4YEBB                234    0.549  79.125  -0.168   0.018   0.019   0.964
B N  4YEBB                235    0.072  10.541  -1.702   0.018   0.088   0.893
E H  4YEBB                236    0.404  73.488   0.977   0.021   0.279   0.699
B I  4YEBB                237    0.040   7.326  -1.094   0.005   0.336   0.660
E N  4YEBB                238    0.451  65.997   0.595   0.004   0.420   0.576
E R  4YEBB                239    0.501 114.660  -0.143   0.005   0.262   0.733
B V  4YEBB                240    0.129  19.827   0.586   0.004   0.085   0.910
E P  4YEBB                241    0.394  55.880   0.315   0.018   0.019   0.964
E P  4YEBB                242    0.493  69.914   0.004   0.782   0.003   0.216
E N  4YEBB                243    0.629  92.027  -0.395   0.694   0.003   0.303
B A  4YEBB                244    0.134  14.723  -0.510   0.694   0.003   0.303
B F  4YEBB                245    0.064  12.825  -1.341   0.694   0.003   0.303
E S  4YEBB                246    0.473  55.424   0.489   0.406   0.004   0.590
E Y  4YEBB                247    0.564 120.591   0.864   0.176   0.004   0.820
B L  4YEBB                248    0.051   9.375   0.543   0.058   0.017   0.925
E R  4YEBB                249    0.545 124.691   1.141   0.053   0.043   0.903
E Q  4YEBB                250    0.407  72.619   1.726   0.053   0.043   0.903
B L  4YEBB                251    0.052   9.613   0.309   0.052   0.084   0.864
E Y  4YEBB                252    0.332  70.884   0.992   0.020   0.205   0.775
E R  4YEBB                253    0.404  92.516   1.607   0.021   0.756   0.223
B L  4YEBB                254    0.038   6.885   0.514   0.018   0.846   0.136
E D  4YEBB                255    0.286  41.184   0.798   0.021   0.756   0.223
B M  4YEBB                256    0.045   9.045  -0.663   0.021   0.279   0.699
E S  4YEBB                257    0.395  46.306  -0.313   0.018   0.088   0.893
E N  4YEBB                258    0.585  85.644  -0.533   0.018   0.047   0.935
B N  4YEBB                259    0.108  15.767  -0.787   0.018   0.088   0.893
E N  4YEBB                260    0.455  66.583   1.081   0.021   0.279   0.699
B L  4YEBB                261    0.050   9.173  -0.384   0.022   0.359   0.619
E S  4YEBB                262    0.425  49.751   0.526   0.004   0.420   0.576
E N  4YEBB                263    0.484  70.799  -0.230   0.005   0.262   0.733
B L  4YEBB                264    0.122  22.265   0.967   0.018   0.088   0.893
E P  4YEBB                265    0.347  49.282   0.384   0.058   0.017   0.925
E Q  4YEBB                266    0.517  92.426   0.086   0.694   0.003   0.303
E G  4YEBB                267    0.632  49.762  -0.687   0.694   0.003   0.303
B I  4YEBB                268    0.125  23.144  -0.882   0.600   0.003   0.397
B F  4YEBB                269    0.103  20.732  -1.694   0.600   0.003   0.397
E D  4YEBB                270    0.524  75.436  -0.148   0.406   0.004   0.590
E D  4YEBB                271    0.532  76.647   0.320   0.176   0.004   0.820
B L  4YEBB                272    0.066  12.011  -0.069   0.058   0.017   0.925
E D  4YEBB                273    0.548  78.895   0.441   0.058   0.017   0.925
E N  4YEBB                274    0.395  57.828   1.299   0.018   0.047   0.935
B I  4YEBB                275    0.051   9.490   0.271   0.018   0.088   0.893
E T  4YEBB                276    0.332  46.062   0.988   0.021   0.279   0.699
E Q  4YEBB                277    0.362  64.600   1.764   0.001   0.900   0.099
B L  4YEBB                278    0.029   5.255   0.849   0.011   0.918   0.071
B I  4YEBB                279    0.239  44.234   1.012   0.011   0.918   0.071
B L  4YEBB                280    0.036   6.555  -0.608   0.022   0.552   0.426
E R  4YEBB                281    0.373  85.532  -0.418   0.004   0.138   0.858
E N  4YEBB                282    0.638  93.403  -0.490   0.005   0.015   0.979
B N  4YEBB                283    0.064   9.296  -0.425   0.005   0.045   0.951
E P  4YEBB                284    0.432  61.301   0.603   0.021   0.279   0.699
B W  4YEBB                285    0.073  17.556  -1.291   0.004   0.420   0.576
E Y  4YEBB                286    0.459  98.174   0.630   0.004   0.514   0.481
B C  4YEBB                287    0.096  13.436  -1.335   0.004   0.514   0.481
E G  4YEBB                288    0.326  25.664  -0.569   0.005   0.262   0.733
B C  4YEBB                289    0.067   9.379  -0.190   0.018   0.088   0.893
E K  4YEBB                290    0.534 109.864   0.332   0.053   0.043   0.903
B M  4YEBB                291    0.126  25.253  -1.764   0.339   0.016   0.645
E K  4YEBB                292    0.340  69.897  -0.160   0.694   0.003   0.303
B W  4YEBB                293    0.300  72.102  -1.404   0.694   0.003   0.303
B V  4YEBB                294    0.072  11.082  -1.634   0.782   0.003   0.216
B R  4YEBB                295    0.192  43.876  -0.101   0.858   0.002   0.139
E D  4YEBB                296    0.503  72.425   0.929   0.858   0.002   0.139
B W  4YEBB                297    0.140  33.766   0.335   0.858   0.002   0.139
B L  4YEBB                298    0.045   8.276  -0.521   0.858   0.002   0.139
E Q  4YEBB                299    0.400  71.494   0.563   0.879   0.010   0.111
E S  4YEBB                300    0.429  50.337   0.295   0.802   0.014   0.185
B L  4YEBB                301    0.151  27.666   0.280   0.622   0.015   0.363
E P  4YEBB                302    0.381  54.064  -0.630   0.430   0.016   0.555
B V  4YEBB                303    0.187  28.711  -1.482   0.268   0.043   0.689
E K  4YEBB                304    0.536 110.296  -0.703   0.184   0.043   0.773
E V  4YEBB                305    0.365  56.147  -1.526   0.113   0.043   0.844
B N  4YEBB                306    0.316  46.248  -1.394   0.053   0.043   0.903
E V  4YEBB                307    0.400  61.480  -1.674   0.053   0.043   0.903
E R  4YEBB                308    0.517 118.462  -1.798   0.053   0.043   0.903
E G  4YEBB                309    0.525  41.357  -1.559   0.053   0.043   0.903
B L  4YEBB                310    0.266  48.650  -1.241   0.052   0.084   0.864
E M  4YEBB                311    0.330  66.093   0.446   0.018   0.088   0.893
B C  4YEBB                312    0.099  13.914  -1.679   0.018   0.088   0.893
E Q  4YEBB                313    0.579 103.392  -0.968   0.018   0.047   0.935
E A  4YEBB                314    0.409  45.061  -0.538   0.018   0.019   0.964
B P  4YEBB                315    0.119  16.829  -0.585   0.257   0.016   0.727
E E  4YEBB                316    0.570  99.596  -0.339   0.321   0.003   0.675
E K  4YEBB                317    0.562 115.542   0.559   0.246   0.004   0.750
B V  4YEBB                318    0.079  12.204  -0.906   0.257   0.016   0.727
E R  4YEBB                319    0.567 129.889   0.470   0.115   0.016   0.868
E G  4YEBB                320    0.785  61.779   0.421   0.018   0.019   0.964
B M  4YEBB                321    0.152  30.415   1.519   0.019   0.141   0.840
E A  4YEBB                322    0.452  49.777   0.426   0.005   0.336   0.660
B I  4YEBB                323    0.254  46.935   0.176   0.021   0.756   0.223
B K  4YEBB                324    0.167  34.372   0.553   0.018   0.846   0.136
E D  4YEBB                325    0.340  48.994  -0.136   0.023   0.655   0.322
B L  4YEBB                326    0.037   6.811  -0.011   0.005   0.262   0.733
E S  4YEBB                327    0.412  48.298   0.990   0.018   0.047   0.935
E A  4YEBB                328    0.557  61.403  -0.677   0.018   0.019   0.964
E E  4YEBB                329    0.363  63.434  -1.106   0.018   0.019   0.964
E L  4YEBB                330    0.480  87.925   0.163   0.018   0.088   0.893
B F  4YEBB                331    0.057  11.460  -0.932   0.020   0.205   0.775
E D  4YEBB                332    0.469  67.626  -0.333   0.022   0.359   0.619
B C  4YEBB                333    0.247  34.637  -1.366   0.004   0.197   0.799
E K  4YEBB                334    0.528 108.548  -0.378   0.004   0.085   0.910
E D  4YEBB                335    0.350  50.363  -0.430   0.018   0.019   0.964
E S  4YEBB                336    0.395  46.329  -0.592   0.257   0.016   0.727
E G  4YEBB                337    0.646  50.872  -1.078   0.430   0.016   0.555
B I  4YEBB                338    0.218  40.404  -0.623   0.430   0.016   0.555
B V  4YEBB                339    0.075  11.512  -1.414   0.339   0.016   0.645
E S  4YEBB                340    0.493  57.733  -0.605   0.257   0.016   0.727
E T  4YEBB                341    0.486  67.380   0.115   0.181   0.016   0.803
B I  4YEBB                342    0.075  13.949   0.017   0.053   0.043   0.903
E Q  4YEBB                343    0.569 101.588   0.129   0.113   0.087   0.800
E I  4YEBB                344    0.442  81.844   1.174   0.056   0.142   0.802
B T  4YEBB                345    0.075  10.375  -0.381   0.056   0.142   0.802
E T  4YEBB                346    0.395  54.856   0.197   0.020   0.205   0.775
E A  4YEBB                347    0.388  42.714   0.843   0.022   0.552   0.426
B I  4YEBB                348    0.049   9.046   0.087   0.022   0.552   0.426
E P  4YEBB                349    0.345  48.984  -0.519   0.022   0.359   0.619
B N  4YEBB                350    0.113  16.616  -1.985   0.018   0.088   0.893
E T  4YEBB                351    0.438  60.681  -1.066   0.018   0.047   0.935
E A  4YEBB                352    0.547  60.301  -1.065   0.018   0.019   0.964
B Y  4YEBB                353    0.067  14.403  -1.560   0.018   0.047   0.935
E P  4YEBB                354    0.478  67.814  -0.331   0.018   0.088   0.893
B A  4YEBB                355    0.105  11.615  -2.273   0.019   0.141   0.840
E Q  4YEBB                356    0.511  91.300  -0.674   0.004   0.197   0.799
E G  4YEBB                357    0.345  27.175  -1.569   0.004   0.138   0.858
E Q  4YEBB                358    0.533  95.122  -1.271   0.004   0.085   0.910
B W  4YEBB                359    0.174  41.775   0.128   0.005   0.015   0.979
B P  4YEBB                360    0.168  23.839  -1.071   0.003   0.003   0.994
E A  4YEBB                361    0.340  37.501  -1.237   0.003   0.003   0.994
E P  4YEBB                362    0.469  66.622  -1.635   0.005   0.015   0.979
B V  4YEBB                363    0.065  10.021  -0.407   0.005   0.045   0.951
B T  4YEBB                364    0.271  37.588  -0.941   0.004   0.138   0.858
B L  4YEBB                365    0.062  11.371  -0.312   0.004   0.420   0.576
E E  4YEBB                366    0.363  63.329   0.969   0.003   0.718   0.279
B V  4YEBB                367    0.149  22.901  -0.906   0.003   0.718   0.279
B L  4YEBB                368    0.202  37.005  -0.016   0.023   0.655   0.322
B F  4YEBB                369    0.144  28.800  -1.172   0.022   0.552   0.426
E Q  4YEBB                370    0.702 125.467   1.165   0.003   0.003   0.994

Explain the output.  Go back.
```

---
